# Hand Eczema and Facial Skin Problems – Association with Occupational Exposures among Community Care Personnel in Sweden: A Cross-sectional Study

**DOI:** 10.2340/actadv.v105.43771

**Published:** 2025-08-03

**Authors:** Thanisorn SUKAKUL, Nils HAMNERIUS, Tina LEJDING, Kajsa Davidson KÄLLBERG, Anna JOSEFSON, Ebba DETLOFSSON, Cecilia SVEDMAN

**Affiliations:** 1 Department of Occupational and Environmental Dermatology, Lund University, Skåne University Hospital, Malmö; 2 Department of Dermatology and Venereology, Örebro University, Örebro University Hospital, Örebro, Sweden

**Keywords:** hand eczema, face, healthcare personnel, epidemiology, handwashing, contact dermatitis

## Abstract

Hand eczema and facial skin problems are common occupational-related skin diseases. However, the data regarding care workers in community care settings are limited. To assess the prevalence and factors associated with hand eczema and facial skin problems among community care personnel, an online questionnaire link was sent to 10,194 personnel in Sweden, with questions regarding hygiene routines, skin problems, and demographics of the participants. Respondents were categorized into groups regarding their skin symptoms. In all, 1,923 (18.9%) responded (89.8% females; 75.9% assistant nurses and care assistants). The 1-year prevalence of hand eczema and facial skin problems was 34.7% and 45.5%, respectively. Dose-dependent associations were found between occupational exposure to soap and water and hand eczema, and duration of face mask use and facial problems. Also, a higher perceived level of stress, female sex, atopic dermatitis, and lower age group were associated with both hand eczema and facial skin problems. In conclusion, healthcare workers in community care have an increased risk of occupationally related skin symptoms, foremost hand eczema, but also facial symptoms related to the use of face masks. Thus, efforts to reduce the harmful effects from the risk factors should be the main concern.

## SIGNIFICANCE

Work-related hand eczema and facial skin problems are of concern not only in healthcare workers but also in community care workers. Occupational exposure to soap, water, and face masks is significant, dose-dependent factors contributing to these issues. Efforts should be directed at reducing the harmful effects of hygiene procedures and protective equipment both in community care and healthcare work to ensure the well-being of those dedicated to caring for others.

Hand eczema is one of the most common occupational skin diseases, often with a multifactorial aetiology, and can affect all age groups (1–5). Hand eczema-related symptoms with relapsing or chronic symptoms have proved to negatively impact the patient’s quality of life (6). The prevalence of hand eczema was previously reported in about 10% of the general population and differed between studies conducted in different periods and countries (6–9). In the general Danish population, the 1-year standardized period prevalence was found to be 4.2% in 2023, which was lower than previously reported from 2021 (13.3%) (6, 7). Previously, persistent hand eczema has been found in 12% of 868 patients with established hand eczema in a general Swedish population (8).

A recently published meta-analysis study reported a 1-year hand eczema prevalence of 27.4% among healthcare workers, which was not significantly different between genders (10). Several studies have shown that healthcare personnel have a high risk of occupationally related hand eczema, particularly due to irritation from wet work exposure (5, 10–12). The vast impact of irritation was clearly shown during the pandemic when the number of healthcare personnel reporting hand eczema increased significantly and where the association between heavier exposure to gloves and hand washing and self-reported hand eczema was identified (3). A particular concern with hand eczema in healthcare workers is the possible risk of infection transmission, as hand eczema can increase the carriage of pathogenic microbes (13).

Healthcare workers are also at risk of having facial skin problems. Facial skin problems caused by personal facial protective equipment have been reported mainly due to irritant contact dermatitis, acneiform eruption, and contact urticaria, while allergic contact dermatitis appears to be rare (14, 15). Compared with hand eczema, signs and symptoms on facial skin can be more challenging to self-evaluate. According to previous publications, facial dermatitis concerning contact dermatitis was the most common diagnosis given to the cases reported although patch testing was not performed, or the results were negative (14, 15). The prevalence of facial skin problems was high during the outbreak of COVID-19, especially among the first-line healthcare workers who wore facial protection equipment (3, 16).

Most of the aforementioned studies focused mainly on the problems among healthcare personnel working in hospital settings. Community care personnel working in ordinary homes or homes for the elderly (community care services) have not been included in the studies performed. In Sweden, more than 100,000 people work as healthcare workers in community care, and the majority (about two-thirds) of assistant nurses and care assistants are employed for community care services (17, 18). Studies on occupational skin disease in these community care workers are scarce (19), and occupational-related skin problems might have been overlooked. An effective skincare and protection routine is essential for both preventing and treating hand eczema, and should ideally be based on evidence-based recommendations. Therefore, this study aimed to investigate the occurrence of hand eczema and facial skin problems and possible associations with occupational skin exposures in community care personnel in Sweden in order to raise awareness of hand eczema, facial skin problems, and the need for prevention.

## MATERIALS AND METHODS

### Questionnaire and participants

This questionnaire-based study was performed in 2022–2023, during and at the end of the COVID-19 pandemic, to survey skin exposures to hygiene procedures and personal protective equipment and the occurrence of hand and face skin disease in community care personnel. A link to the survey was distributed by email to all community care personnel employed in 4 municipalities in Örebro, Malmö, Karlskrona, and Halmstad in Sweden. The electronic questionnaire was delivered, and the responses were collected via an online survey tool, SUNET (the Swedish Research Council, organization number 2021005208, Stockholm, Sweden). The study was approved by the Swedish Ethical Review Authority (Dnr 2021-01596). The personnel who agreed to participate in this study consented to respond to the questionnaire before answering the question.

The questionnaire included participants’ demographics, risk exposure possibly causing hand eczema and facial skin problems such as hygiene procedures and the use of protective equipment at work and in leisure time, history of hand eczema, facial skin signs, and symptoms.

### Statistical analysis

Statistical analysis was performed in IBM SPSS Statistics for Windows (version 29.0; IBM Corp, Armonk, NY, USA). No sample size calculations were made prior to the main analyses of the questionnaire study. The demographics of respondents and the prevalence of self-reported hand eczema and facial skin problems were analysed using descriptive methods including mean (standard deviation) for age or proportion, reported as percentage for others. Missing data and individual “unknown” responses were excluded from the analysis. The raw data from the questionnaire might be categorized into categorical outcomes, which could be binary, or ordinal as shown in the result tables below. Comparisons were performed between respondents with and without skin disease and between occupations engaged in patient care (assistant nurses and care assistants) and other occupations (nurses, physiotherapists, occupational therapists).

Pearson’s χ^2^ test was used to demonstrate the associations between groups with 2 or more categories. When comparing factors in an ordinal scale, *p*-values for trend (linear-by-linear association) were reported. Univariable logistic regression was performed to demonstrate crude odds ratios (OR) of the factors that could be associated with self-reported hand eczema or facial skin problems, while multivariable logistic regression analysis was further performed by including the factors with a *p*-value less than 0.2 according to the univariable logistic regression to report the adjusted ORs.

## RESULTS

### Respondents

**Fig. 1:**
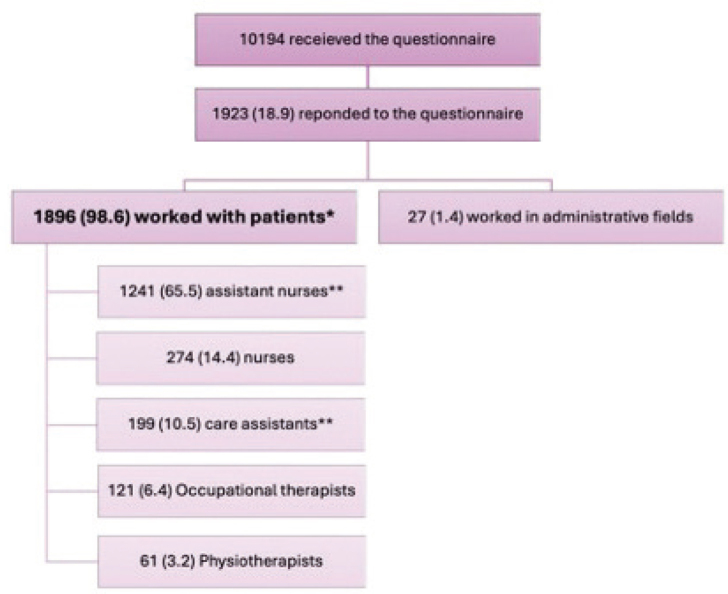
**Respondents and occupations.** *Total participants included in the analysis. **Assistant nurses and care assistants were expected to work closely with the patients (total n = 1,440, 75.9%).

The electronic questionnaire was sent to 10,194 employees via email, of which 1,923 (18.9%) responded to the questionnaire (**Fig. 1**). The respondents who worked exclusively with the administration were excluded (*n* = 27). Therefore, 1,896 (98.6%) were included in the statistical analysis as they reported working with patients.

Most of the respondents were female (89.8%). The mean age (standard deviation) was 47.2 (12.0) years, ranging from 17 to 69 years. A history of atopic dermatitis (AD) was reported by 20.6% of the respondents. The majority of the responders were assistant nurses (65.5%). The respondents engaged in direct patient care work, assistant nurses and care assistants, accounted for 75.9%, while the remaining group consisted of nurses, physiotherapists, and occupational therapists.

### Hand eczema and occupational skin exposures

Compared with before the pandemic, an increase in occupational exposure to soap and water was reported by 59%, alcoholic hand disinfectants by 73%, and disposable gloves by 38% (**Table I**). The 1-year prevalence of self-reported hand eczema was 34.7% (657/1896) and the point prevalence was 20.7% (392/1896). Of those with hand eczema, 85.9% reported improvement during days off work or vacation. About one-fifth (22.3%) had visited a doctor and 1.4% had been on sick leave due to hand eczema.

**Table I:** Self-reported change in occupational skin exposure during the COVID-19 pandemic stratified on occupational groups

Change in occupational skin exposure during the pandemic compared with before the pandemic	All participants (*n* = 1,896)		Assistant nurse and care assistant (*n* = 1,440)		Nurse and occupational therapist and physiotherapist (*n* = 456)	
	*n*	%	*n*	%	*n*	%
More frequent handwashing with water and soap	1,127	59.4	847	58.8	280	61.4
More frequent alcoholic hand disinfectant use	1,382	72.9	1,026	71.3	356	78.1
More frequent glove use	720	38.0	561	39.0	159	34.9
More frequent facial mask use	1,508	79.5	1,095	76.0	413	90.6

**Table II** demonstrates factors associated with self-reported hand eczema within the previous 12 months. In univariable logistic regression analysis, there were dose-dependent associations between hand eczema and exposures to soap and water, alcoholic hand disinfectant, and disposable gloves, respectively, but in the multivariable logistic regression analysis, only the dose-dependent association between hand eczema and soap and water was significant. Adjusted ORs (95% confidence interval, CI) for respondents who washed their hands 11–20 times and more than 20 times were 1.63 (1.15–2.29) and 2.28 (1.55–3.37), respectively, compared with those who washed their hands 10 times per day or less.

**Table II:** Factors associated with self-reported hand eczema during the past 12 months

Factors	Total		Hand eczema during the past 12 months				Univariable analysis		Multivariable analysis (*n* = 1,396)	
			No		Yes					
	*n*	%	*n*	%	*n*	%	*p*-value	OR (95 CI)*	*p*-value	OR (95 CI)*
*Exposure at work*										
Handwashing with water and soap (times per day)	1896		1239		657		<0.001**		<0.001	
0–10	504	26.6	372	30.0	132	20.1		1		1
11–20	666	35.1	442	35.7	224	34.1		1.43 (1.11–1.84)		1.63 (1.15–2.29)
> 20	726	38.3	425	34.3	301	45.8		2.00 (1.56–2.56)		2.28 (1.55–3.37)
Use of alcohol hand disinfectant (times per day)	1896		1239		657		<0.001**		0.113	
0–20	352	18.6	265	21.4	87	13.2		1		1
21–50	791	41.7	540	43.6	251	38.2		1.42 (1.07–1.88)		1.23 (0.84–1.81)
> 50	753	39.7	434	35.0	319	48.6		2.24 (1.69–2.97)		1.54 (1.00–2.35)
Glove use (hours per day)	1896		1239		657		<0.001**		0.501	
<1	394	20.8	273	22.0	121	18.4		1		1
1–3	528	27.8	374	30.2	154	23.4		0.93 (0.70–1.24)		0.88 (0.61–1.25)
> 3	974	51.4	592	47.8	382	58.1		1.46 (1.13–1.87)		1.05 (0.74–1.48)
*Exposure after work*										
Handwashing with water and soap (times per day)	1896		1239		657		<0.001**		0.930	
0–10	873	46.0	602	48.6	271	41.2		1		1
11–20	745	39.3	476	38.4	269	40.9		1.26 (1.02–1.54)		1.05 (0.80–1.38)
> 20	278	14.7	161	13.0	117	17.8		1.61 (1.22–2.13)		1.06 (0.69–1.61)
Use of alcohol hand disinfectant (times per day)	1891		1234		657		0.189		0.419	
0–20	1595	84.3	1050	85.1	545	83.0		1		1
21–50	198	10.5	125	10.1	73	11.1		1.13 (0.83–1.53)		0.78 (0.51–1.18)
> 50	98	5.2	59	4.8	39	5.9		1.27 (0.84–1.93)		0.78 (0.43–1.42)
*General demographics*										
Age group (year)	1889		1235		654		<0.001**		0.004	
18–29	193	10.2	113	9.1	80	12.2		1		1
30–39	347	18.4	187	15.1	160	24.5		1.21 (0.85–1.73)		1.25 (0.79–1.97)
40–49	426	22.6	278	22.5	148	22.6		0.75 (0.53–1.07)		0.87 (0.55–1.37)
50–59	596	31.6	414	33.5	182	27.8		0.62 (0.44–0.87)		0.67 (0.43–1.04)
60+	327	17.3	243	19.7	84	12.8		0.49 (0.33–0.71)		0.58 (0.35–0.96)
Gender	1893		1236		657		0.016		0.044	
Female	1703	90.0	1097	88.8	606	92.2		1.51 (1.08–2.11)		1.60 (1.01–2.53)
Male	190	10.0	139	11.2	51	7.8		1		1
History of atopic dermatitis	1723		1127		657		<0.001		<0.001	
No	1333	77.4	927	82.3	406	68.1		1		1
Yes	390	22.6	200	17.7	190	31.9		2.17 (1.72–2.73)		2.06 (1.57–2.71)
Psychosocial aspect										
Number of house residences (person)	1871		1227		644		0.135		0.979	
1–2	1038	55.5	696	56.7	342	53.1		1		1
> 2	833	44.5	531	43.3	302	46.9		1.16 (0.96–1.40)		1.00 (0.76–1.31)
Having children aged less than 4 years	1555		1030		525		0.006		0.327	
No	1330	85.5	899	87.3	431	82.1		1		1
Yes	225	14.5	131	12.7	94	17.9		1.50 (1.12–2.00)		1.20 (0.83–1.74)
Stress level	1887		1235		652		<0.001**		<0.001	
Never or a few times per year	319	16.9	240	19.4	79	12.1		1		1
Once per month	477	25.3	345	27.9	132	20.2		1.16 (0.84–1.61)		1.06 (0.72–1.55)
Once per week	420	22.3	282	22.8	138	21.2		1.49 (1.07–2.06)		1.44 (0.98–2.12)
A few times per week	391	20.7	226	18.3	165	25.3		2.22 (1.60–3.07)		2.03 (1.36–3.01)
Almost everyday	280	14.8	142	11.5	138	21.2		2.95 (2.09–4.17)		2.54 (1.66–3.91)
Occupation group	1896		1239		657		0.002		0.795	
Assistant nurse/care assistant	1440	75.9	914	73.8	526	80.1		1.43 (1.14–1.80)		0.96 (0.70–1.32)
Nurse/physiotherapist/occupational therapist	456	24.1	325	26.2	131	19.9		1		1

Variables included in the multivariable analysis are the variables with a *p*-value equal to or less than 0.2 demonstrated by the univariable analysis;

* *P*-value for trend.

According to the multivariable logistic regression analysis, respondents in the age groups from 18 to 59 years were at a similar risk of having hand eczema, unlike the respondents aged equal to or more than 60 years who had a significantly lower risk of having hand eczema. Hand eczema was significantly more frequent in women and respondents with a history of AD. Having a higher level of self-reported stress was significantly associated with hand eczema.

**Fig. 2:**
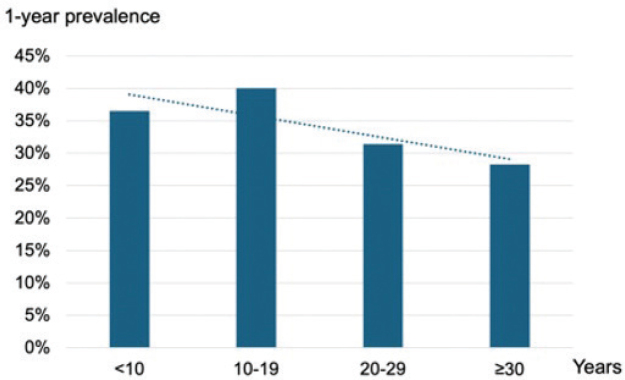
**Hand eczema prevalence in relation to number of years working in community care.** P-value for trend = 0.002.

Regarding work experience, the number of years working in community care was used as a proxy for work experience. In total, there was a significant trend for a lower 1-year prevalence of hand eczema with an increasing number of years working in community care (**Fig. 2**). However, there was no significant difference in prevalence among those who had worked less than 5 years compared with 5–10 years in community care (33% [115/344] vs 39% [166/426], *p*-value = 0.11).

### Facial skin problems and occupational skin exposures

Compared with before the pandemic, an increase in occupational exposure to face masks was reported by 79% (see Table I). The 1-year prevalence of self-reported facial skin problems was 45.5% (862 respondents) and 618 (point prevalence 32.6%) of them had had recent problems with their facial skin. The most common skin problem reported was dryness (70.0%), followed by redness (52.0%), itchiness (39.1%), vesicles or pus (29.8%), erosion or ulcer (11.1%), and other unspecified symptoms (17.6%).

Factors associated with self-reported facial skin problems during the past 12 months are demonstrated in **Table III**. Similar to hand eczema, facial skin problems were significantly more common in lower age groups than in older groups. Facial skin problems were significantly associated with being female and having a history of AD.

**Table III:** Factors associated with self-reported facial skin problems during the past 12 months

Factors	Total		Facial skin problems during the past 12 months				Univariable analysis		Multivariable analysis (*n* = 1,401)	
			No		Yes					
	*n*	%	*n*	%	*n*	%	*p*-value	OR (95 CI)	*p*-value	OR (95 CI)
*Exposure at work*										
Face mask use (hours per day)	1896		1034		862		<0.001*		<0.001	
<2	492	25.9	315	30.5	177	20.5		1		1
2–5	550	29.0	313	30.3	237	27.5		1.35 (1.05–1.73)		1.73 (1.27–2.35)
> 5	854	45.0	406	39.3	448	52.0		1.96 (1.56–2.47)		2.57 (1.93–3.45)
Face shield use (hours per day)	1884		1027		857		0.395*			
0	754	40.0	429	41.8	325	37.9		1		NA
0.1–1	505	26.8	255	24.8	250	29.2		1.29 (1.03–1.62)		NA
> 1	625	33.2	343	33.4	282	32.9		1.09 (0.88–1.34)		NA
*Exposure after work*										
Face mask use	1885		1027		858		0.160		0.004	
No	1061	56.3	563	54.8	498	58.0		1		1
Yes	824	43.7	464	45.2	360	42.0		0.88 (0.73–1.05)		0.71 (0.56–0.90)
*General demographics*										
Age group (year)	1889		1029		860		<0.001*		<0.001	
18–29	193	10.2	82	8.0	111	12.9		1		1
30–39	347	18.4	170	16.5	177	20.6		0.77 (0.54–1.10)		0.77 (0.49–1.19)
40–49	426	22.6	228	22.2	198	23.0		0.64 (0.46–0.90)		0.62 (0.40–0.96)
50–59	596	31.6	339	32.9	257	29.9		0.56 (0.40–0.78)		0.49 (0.32–0.76)
60+	327	17.3	210	20.4	117	13.6		0.41 (0.29–0.59)		0.36 (0.23–0.58)
Gender	1896		1034		862		<0.001		<0.001	
Female	1703	90.0	889	86.1	814	94.5		2.79 (1.98–3.92)		3.12 (1.99–4.90)
Male	190	10.0	143	13.9	47	5.5		1		1
History of atopic dermatitis	1896		1034		862		<0.001		0.045	
No	1333	77.4	761	80.8	572	73.2		1		1
Yes	390	22.6	181	19.2	209	26.8		1.54 (1.22–1.93)		1.31 (1.01–1.72)
*Psychosocial aspect*										
Number of house residences (person)	1871		1018		853		0.943			
1–2	1038	55.5	564	55.4	474	55.6		1		NA
> 2	833	44.5	454	44.6	379	44.4		0.99 (0.83–1.19)		NA
Having children aged less than 4 years	1555		849		706		0.057		0.007	
No	1330	85.5	713	84.0	617	87.4		1		1
Yes	225	14.5	136	16.0	89	12.6		0.76 (0.57–1.01)		0.62 (0.44–0.88)
Stress level	1887		1031		856		<0.001*		<0.001	
Never or a few times per year	319	16.9	211	20.5	108	12.6		1		1
Once per month	477	25.3	288	27.9	189	22.1		1.28 (0.95–1.72)		1.05 (0.74–1.50)
Once per week	420	22.3	217	21.0	203	23.7		1.83 (1.35–2.47)		1.83 (1.28–2.61)
A few times per week	391	20.7	194	18.8	197	23.0		1.98 (1.46–2.69)		1.74 (1.20–2.53)
Almost every day	280	14.8	121	11.7	159	18.6		2.57 (1.84–3.58)		2.23 (1.48–3.36)
Occupation groups	1896		1034		862		0.772			
Assistant nurse/care assistant	1440	75.9	788	76.2	652	75.6		1.03 (0.84–1.28)		NA
Nurse/physiotherapist/occupational therapist	456	24.1	246	23.8	210	24.4		1		NA

Variables included in the multivariable analysis are the variables with a *p*-value equal to or less than 0.2 demonstrated by the univariable analysis.

* *P*-value for trend. NA: not applicable.

Regarding the risk exposure at work, respondents using face masks for 2 h or more reported significantly more facial skin problems. In contrast, respondents with facial skin problems reported significantly less use of face masks outside of work. The frequency of using face shields did not relate to facial skin problems.

### Different occupation groups

Assistant nurses and care assistants reported having significantly more hand and facial skin exposure compared with other occupations (nurses, physiotherapists, and occupational therapists) (**Table IV**). About half of them washed their hands with water and soap more than 20 times and used alcoholic hand disinfectants more than 50 times. They used significantly more gloves, face masks, and face shields at work than in other occupations; they also had a higher exposure to water and soap and alcoholic hand disinfectant, and wore face masks outside work.

**Table IV:** Exposure stratified on occupation groups

Exposure	Total		Occupation groups				*p*-value
			Assistant nurse and care assistant		Nurse and occupational therapist and physiotherapist		
	*n*	%	*n*	%	*n*	%	
*Hand*							
*Exposure at work*							
Handwashing with water and soap (times per day)	1,896		1,440		456		< 0.001*
0–10	504	26.6	263	18.3	241	52.9	
11–20	666	35.1	501	34.8	165	36.2	
> 20	726	38.3	676	46.9	50	11.0	
Use of alcoholic hand disinfectant (times per day)	1,896		1,440		456		< 0.001*
0–20	352	18.6	179	12.4	173	37.9	
21–50	791	41.7	588	40.8	203	44.5	
> 50	753	39.7	673	46.7	80	17.5	
Glove use (hours per day)	1,896		1,440		456		< 0.001*
< 1	394	20.8	225	15.6	169	37.1	
1–3	528	27.8	318	22.1	210	46.1	
> 3	974	51.4	897	62.3	77	16.9	
Glove use (pairs per day)	1,876		1,422		454		< 0.001*
0–10	342	18.2	85	6.0	257	56.6	
11–20	399	21.3	267	18.8	132	29.1	
> 20	1135	60.5	1070	75.2	65	14.3	
*Exposure after work*							
Handwashing with water and soap (times per day)	1,896		1,440		456		< 0.001*
0–10	873	46.0	601	41.7	272	59.6	
11–20	745	39.3	590	41.0	155	34.0	
> 20	278	14.7	249	17.3	29	6.4	
Use of alcoholic hand disinfectant (times per day)	1891		1435		456		< 0.001*
0–20	849	44.9	581	40.5	268	58.8	
21–50	423	22.4	320	22.3	103	22.6	
> 50	619	32.7	534	37.2	85	18.6	
*Face*							
*Exposure at work*							
Face mask use (hours per day)	1,896		1,440		456		< 0.001*
< 2	492	25.9	353	24.5	139	30.5	
2–5	550	29.0	362	25.1	188	41.2	
> 5	854	45.0	725	50.3	129	28.3	
Face shield use (hours per day)	1,884		1,429		455		0.103*
0	754	40.0	583	40.8	171	37.6	
0.1–1	505	26.8	335	23.4	170	37.4	
> 1	625	33.2	511	35.8	114	25.1	
*Exposure after work*							
Face mask use	1,885		1,432		453		< 0.001
No	1,061	56.3	774	54.1	287	63.4	
Yes	824	43.7	658	45.9	166	36.6	

* *P*-values for trend.

Hand eczema was significantly more common among assistant nurses and care assistants compared with the other occupations (nurses, physiotherapists, and occupational therapists), with 1-year prevalences of 36.5% (526/1440) and 28.7% (131/456), respectively (*p* = 0.002). However, after corrections for other related factors such as occupational exposures in the multivariate logistic regression analysis, there was no significant difference (OR [95% CI] = 0.99 [0.72–1.36]). Facial skin problems were equally common among assistant nurses and care assistants compared with the other occupations, with 1-year prevalences of 45.3% (652/1440) vs 46.1% (210/456), *p*-value = 0.77.

## DISCUSSION

In this study of community care workers, hand eczema is more commonly reported in respondents with high levels of occupational exposure to soap and water, alcoholic hand disinfectants, and disposable gloves, as well as non-occupational exposure to soap and water. However, after multivariate logistic regression analysis, a significant association was found only for occupational exposure to soap and water. The clear association between hand eczema and exposure to hand washing is in line with previous studies in hospital healthcare personnel (3, 5, 20), and 2 extensive systematic reviews where hand washing was a risk factor for irritant contact dermatitis, while this could not be shown for alcoholic hand disinfectants (11, 12). However, there are data indicating that alcoholic hand disinfectant exposure on wet skin can be harmful to the skin (21, 22). In the present study, after multivariable logistic regression analysis, an almost significant association was found when comparing a high level of alcoholic hand disinfectant exposure with low OR (1.54, 95% CI = 1.00–2.35). We do not know if the different work conditions in community care (working in people’s homes and not in a hospital ward or a doctor’s office) could lead to increased use of alcoholic hand disinfectants on wet skin, as this has not been studied.

Even though high exposure (> 3 h per day) to disposable gloves was common in the study group (see Table II), the difference in exposure between those with and those without eczema was not significant. This contrasts with previous studies in hospital healthcare personnel that have shown a significant association between hand eczema and disposable gloves (3, 5, 11, 23), and suggests non-identified confounding factors or possibly underpowered subgroup analyses. Furthermore, increased occupational exposure to gloves during the pandemic was less commonly reported than increased exposure to soap and water (see Table I). Thus, in the present study of community care workers, the more frequent occupational exposure to soap and water was the dominant risk factor for occupational hand eczema. Equally, the influence of non-occupational wet work was limited, and no significant association with hand eczema could be shown in the regression analysis. Furthermore, the majority (86%) reported that their hand eczema improved when off work. The study questions did not attempt to differentiate between different severity levels of hand eczema. However, sick leave because of hand eczema was very rare, which could indicate mild disease. On the other hand, more than one-fifth had consulted a doctor for their hand eczema, which indicates not so mild disease, and one cannot exclude that presenteeism in part can explain the low level of sick leave.

Occupations engaged in patient care (assistant nurses and care assistants) reported hand eczema more often than other occupations (nurses, physiotherapists, and occupational therapists). However, in the multivariate regression analysis, no statistically significant difference could be shown. Although other factors such as educational level might have an influence, the data indicate that wet work exposure is the main cause of the higher prevalence of hand eczema in the patient care group. This further illustrates the harmful effect of occupational soap and water exposure in community care work.

This study demonstrated significant associations between hand eczema and age, sex, and history of AD, which are well-recognized risk factors for hand eczema (24, 25). Young age can reflect more household wet work exposure, for example, care of small children. Lower hand eczema prevalence in older age groups can indicate a healthy worker effect where workers who have experienced hand eczema have changed occupations. On the other hand, hand eczema in young age groups could be related to less experience in work. Incidence data show that hand eczema mostly arises during the first period of occupation and the risk could decline thereafter (26). However, no support for this was found in the present study, where a significantly lower hand eczema prevalence was seen only in those with > 30 years of community care experience. As expected, hand eczema was associated with a history of AD, with an OR of about 2, thus in this study comparable to the OR found for high exposure to soap and water. However, the figure is lower than what has been reported previously in an extensive systematic review and meta-analysis (OR [95% CI] = 4.29 [3.13–5.88]) (24). There was a dose-dependent association between stress and hand eczema, which has been reported in other hand eczema studies (27). Hand eczema does influence quality of life, but the possible role of stress as an aggravating factor for hand eczema has also been discussed (27).

In community care workers, there was a dose-dependent association between daily time using face masks and experiencing facial skin problems, which is in line with studies in hospital healthcare workers (3, 28, 29). Equally, the spectrum of reported symptoms is in line with studies in healthcare workers (30). It is very likely that facial skin problems became more prevalent during the pandemic, as there was a statistically significant association between face mask use and reporting facial skin problems, and the majority of the respondents reported increased exposure to face masks during the pandemic. However, one should be aware that the data on the prevalence of facial skin problems before the pandemic are very limited and no reliable comparisons can be made.

There is a risk of potential bias and imprecision in this study. Not having Swedish as a native language can be a cause for non-participation or imprecision. Recall bias can affect the prevalence of disease, as well as confounding factors, and it has been shown that the prevalence of hand eczema could be underestimated, while the prevalence of childhood eczema can also be underestimated (31). Furthermore, given the high number of non-responders, there is an obvious risk of selection bias and the prevalence figures must be interpreted with caution. However, such influence on the association analyses would be dependent on a skewed reporting of both skin disease and exposures, which is less likely, and therefore the association analyses are more robust.

The reported 1-year prevalences of hand eczema and facial skin problems in the responders were 35% and 45%, respectively. But if the 1-year-prevalence among the non-responders was only half the prevalences reported by the responders, the overall prevalences would be 21% and 27%, respectively. Theoretically, although less likely, the prevalence among non-responders could be higher than among responders. In a large systematic review including studies both before and during the COVID-19 pandemic, the pooled 1-year prevalence of hand eczema in healthcare workers was 27.4% (95% CI = 19.3–36.5) (10). The pooled overall prevalence (whether point, 1-year, or lifetime prevalence not specified) of facial dermatoses was 55% in a large systematic review that included studies in the general population as well as in healthcare workers (29), while the 1-year prevalence of facial skin disease in hospital healthcare workers in southern Sweden was 23%. Thus, the prevalences reported show that both hand eczema and facial skin problems are frequent in community care workers and should be regarded with the same concern as in healthcare workers.

In conclusion, this study, conducted during and immediately after the COVID-19 pandemic, demonstrates that hand eczema and facial skin problems are common among community care personnel and that occupational skin exposures to soap and water and face masks, respectively, are major, dose-dependent factors. Improvement of hand eczema in healthcare work, and measures to prevent occupational-related hand eczema and facial skin problems are urgently needed. This should include not only the use of moisturizers but also education on avoidance of excessive handwashing with water and soap and prolonged facial mask use and encouraging the use of alcoholic hand disinfectants.
